# How broad are state physician health program descriptions of physician impairment?

**DOI:** 10.1186/s13011-018-0168-z

**Published:** 2018-08-23

**Authors:** Nicholas D. Lawson, J. Wesley Boyd

**Affiliations:** 1 Georgetown University Law Center, 600 New Jersey Ave NW, Washington, DC, 20001 USA; 2 Department of Psychiatry, Cambridge Health Alliance/Harvard Medical School, 26 Central St, Somerville, MA 02143 USA

**Keywords:** Physician impairment, Physician health program, Addiction, Substance use disorder, Coercion, Stigma, Discrimination, Physician suicide, Americans with Disabilities Act, Wellness, Well-being

## Abstract

**Background:**

Physician health program websites in 23 states provide many descriptions of possible physician impairment. This study sought to determine whether these descriptions are so broad that almost everyone might potentially be suspected of being impaired given these descriptions.

**Methods:**

The authors randomly selected 25 descriptions of impairment and then presented them anonymously online to members of the general population in full-time employment through Amazon’s Mechanical Turk (*N* = 199). Half of the respondents randomly received a narrowly worded version, and half received a broadly worded version of the survey questions.

**Results:**

In the narrowly worded version of the survey, 70.9% of respondents endorsed at least one description of impairment, and 59.2% endorsed more than one. In the broadly phrased version, 96.9% endorsed at least one description, and 95.8% endorsed more than one. These respondents endorsed a median of 10 out of 25 (40%) descriptions.

**Conclusions:**

These findings call into question whether these descriptions really identify persons with poor performance or who pose a high risk of substantial, imminent harm to self or others in the workplace. They also demonstrate the extent to which these descriptions could potentially be misapplied and brand almost anyone as impaired.

**Electronic supplementary material:**

The online version of this article (10.1186/s13011-018-0168-z) contains supplementary material, which is available to authorized users.

## Background

At present, most physician impairment policies selectively target physicians with physical and mental disorders (including substance use disorders [SUDs]) or disabilities. The American Medical Association (AMA), for example, defines physician impairment exclusively in terms of “physical, mental, or behavioral disorder[s],” [1] and mandates reporting and referrals of those suspected of being impaired to state physician health programs (PHPs) [2]. Some state laws also contain definitions [3] that state that, “‘impaired’ or ‘impairment’ means the presence of the diseases of alcoholism, drug abuse, or mental illness.” (We consider the term *mental disorder* to include SUDs for the remainder of this article unless otherwise stated.)

There is very little evidence to suggest, however, that mental disorders in physicians are a meaningful cause of medical errors or preventable adverse events [4, 5]. On the basis of empirical research, male sex appears to be a better predictor of poor performance or subsequent professional discipline than the presence of a mental disorder [6–12]. Therefore, it is not entirely clear why these policies have persisted and continue to encourage more referrals to PHPs.

State PHPs, which were originally designed primarily to treat physicians with SUDs, have expanded their outreach efforts over the years, and many provide descriptions of impairment or reasons to refer physicians to their programs that appear very broad. These descriptions are also regularly reinforced at hospital orientations, conferences, and other educational settings, which raises concerns about inappropriate targeting and mislabeling of physicians as impaired.

### State PHP descriptions of impairment

Many PHPs list, on their websites or publications otherwise, signs of behavior by physicians that are potentially concerning for either a substance use or other mental disorder. Some PHPs state that some of their descriptions of impairment are not specific and may not necessarily indicate impairment, though one [13] has described the presence of more than one sign or symptom as likely to be “a reliable indicator.” All told, lists of “possible reasons for referral” or “indicators of impairment” are posted on the PHP websites of 23 states (including the District of Columbia).

Thus far, however, no empirical research has evaluated whether the presence of one, two, or more of these signs and symptoms really are reliable indicators of impairment. Given a superficial examination of these descriptions (see Additional file 1 for complete descriptions, weblinks, and other context), it is hard not to wonder whether they might actually describe behaviors or conditions exhibited by most physicians and even most members of the general public at various points in their lives. Research has yet to determine whether the published lists by PHPs are so broad that almost everyone could potentially be labeled as impaired.

Additionally, it is often unclear whether or not PHP descriptions of impairment are meant to apply to current or recent behaviors, or else to events from the distant past. Since state medical boards have been criticized for asking broad questions about a history of mental health treatments, without reference to timeframe, on medical license applications [14], this is an important consideration.

Many PHP descriptions also do not indicate whether they are meant to apply only to work settings or whether they should apply to other settings as well. Physician-employees might read one of these descriptions and assume that it applies only to behavior at work. But state PHPs and addiction providers, who sometimes justify coercive investigations into spousal and other family concerns [15], might apply them to family, community, and other domains outside the context of work.

In light of this, it cannot always be assumed that these descriptions will be applied in predicable, responsible, and appropriate ways. State medical board executive directors, for example, may have very poor knowledge regarding the laws that apply to reporting physicians for examinations and imposing disciplinary sanctions. To wit, one survey of state medical board executive directors [16] found that “thirteen of the 35 [state medical board executive directors responding to the survey, or 37%] indicated that [they believed that] the diagnosis of mental illness by itself was sufficient for sanctioning physicians.”

Given the concerns these state medical board directors’ answers raise and the very broad nature of many PHP lists of “concerning signs,” we constructed an online survey with both narrowly worded and broadly phrased versions of questions for members of the general public in full-time employment to find out how many of these descriptions they would endorse. The narrowly phrased version provided a conservative estimate of respondents’ endorsements, and the broadly phrased version explored the limits of just how far these descriptions could be applied and interpreted. We designed this study and constructed our survey to find out whether these descriptions might be so broad that they could potentially be misapplied and brand almost anyone as impaired.

## Methods

We chose not to survey physicians but rather members of the general population in full-time employment through an online, anonymous survey using Amazon’s Mechanical Turk (MTurk). While the properties and methods of research using MTurk have been studied and described in detail, surveys of physicians seemed more susceptible to response bias and more difficult to carry out in a way that would yield reliable data. We also did not think it necessary to survey physicians specifically in order to test our research hypothesis in general terms. Findings that most of the public could be described by these various signs and symptoms would still call into question whether they serve a meaningful purpose and are susceptible to being overapplied. A complete report of our methods and results in accordance with the Checklist for Reporting Results of Internet E-Surveys (CHERRIES) [17] appears as Additional file 2.

### Amazon’s Mechanical Turk

MTurk is a crowdsurfing market. Crowdsurfing refers to “the distribution of tasks to large groups of individuals via a flexible, open call.” [18] As described by Chandler and Shapiro [17], “Online labor markets, such as [MTurk, are] designed to match people (requesters) requesting the completion of small tasks [referred to here as human intelligence tasks (HITs)] with people willing to do them (workers).... MTurk is currently the dominant crowdsourcing market used by academic researchers.” According to several sources [17–20], “the data obtained [through MTurk] are at least as reliable as those obtained via traditional methods” in social science and psychology research [21], which often rely on in-person interviews and surveys.

MTurk populations are also diverse. Compared to the US population as a whole, MTurk workers are above average in cognitive aptitude, with studies reporting mixed findings on the prevalence of mental disorders [20]. They are somewhat younger, predominantly Caucasian, more educated [17, 19], and 24% report that they are unemployed but would prefer to be employed, compared to 8% of the US population overall [19]. Despite the fact that these respondents probably vary slightly from most in the US along these lines, anonymous online surveys are more likely to elicit honest responses than face-to-face interviews, and more likely to avoid social desirability bias [22], which results when respondents give answers to make the interviewer think positively about them.

### Sample

Two hundred participants were recruited from MTurk under the restriction that they resided in the US; had a task approval rating of at least 95%; had completed at least 1000 prior tasks on MTurk; and were currently employed full-time, working at least 35 h per week. Except for employment, these restrictions are standard for research using MTurk. These qualifications ensured consistency with prior research on MTurk, and high quality of data, since workers who start but do not complete tasks can skew survey results. MTurk participants in general are at least 18 years old.

On MTurk, workers consent to all tasks after being informed of the risks and benefits of participation, and browse tasks by title, keyword, reward, availability, and so on, to complete those that interest them. Only MTurk workers who met our prescreening requirements could view and decide whether to participate in the study. A notice was posted on MTurk requesting workers to “answer a survey about your experiences in work and other settings,” were told that it would take no more than 5 min to complete, and that they would be paid $0.30 electronically for their participation. Respondents were anonymized as soon as they agreed to participate and given a link to complete the survey on a separate website. Their responses were kept separate from potentially identifying information. The Institutional Review Board of Vassar College determined this study was exempt from review.

### Measures

Table 1 lists PHP website descriptions of impairment that were randomly selected for the survey, with both narrow and broad versions of the wording used to construct survey items. Half of the participants were assigned to the narrowly worded version of the survey, and the rest were assigned to the broadly worded version.

**Table 1:** Wording used to survey respondents for endorsement of 25 state physician health program descriptions of impairment

		Specifies if			
	Description	Currently?	At work?	Narrow	Broad
1	Intimidation	No	Yes	Have you intimidated someone at work?	Have you ever intimidated someone at work?
2	A deterioration in personal hygiene	No	No	Has there been deterioration in your personal hygiene?	Has there ever been deterioration in your personal hygiene?
3	Constant sadness or tearfulness	No	No	Have you been constantly sad or tearful?	• Have you ever been constantly sad? • Have you ever been constantly tearful?
4	Occurrence of spouse, child abuse	No	No	Has your spouse or have your children been abused?	• Has your spouse or ex-spouse ever been abused? • Has your child or have your children ever been abused?
5	Easily agitated, irritable	No	No	Have you been easily agitated or irritable?	• Have you ever been easily agitated? • Have you ever been irritable?
6	Increased patient complaints	No	Yes	Have there been increased complaints about your work from clients, customers, or other consumers?	Have there ever been increased complaints from client, customer, or other consumer complaints about your work?
7	Personality and behavioral changes	No	No	Have you had personality and behavioral changes?	Have you ever had personality and behavioral changes?
8	Neglected social commitments	No	No	Have you neglected social commitments?	Have you ever neglected social commitments?
9	DWI arrest or DUI violations	No	No	Have you had a DWI arrest or DUI violation?	• Have you ever been arrested for DWI? • Have you ever had a DUI violation?
10	Other mental health concerns that directly impact work performance	No	Yes	Have you had mental health concerns that directly impact work performance?	Have you ever had mental health concerns that directly impacted your performance at work?
11	Direct statements indicating distress	No	No	Have you made direct statements indicating distress?	Have you ever made direct statements indicating distress?
12	If the resident is experiencing problems coping with patients or with the typical stress of a busy residency	Yes	No	Are you experiencing problems coping with clients, customers, or other consumers at work, or with the typical stress of a busy job?	• Have you ever had problems coping with clients, customers, or other consumers? • Have you ever had problems coping with the typical stress of a busy job?
13	Sweating when otherwise comfortable	No	No	Have you been sweating when otherwise comfortable?	Have you ever been sweating while otherwise comfortable?
14	Tremors, hands shake	No	No	Have you had tremors or hands shake?	Have you ever had tremors or hands shake?
15	Rapid or pressured speech	No	No	Have you had rapid speech?	Has your speech ever been rapid?
16	Hospital personnel question competence and/or behavior	Yes	Yes	Do personnel at work question your competence and/or behavior?	• Have work personnel ever questioned your competence? • Have work personnel ever questioned your behavior?
17	Makes degrading or demeaning comments regarding patients, families, nurses, physicians, hospital personnel, or the hospital. The physician’s non-constructive criticism often works to intimidate, undermine confidence, belittle, or imply stupidity or incompetence in his or her victims.	Yes	No	Do you make demeaning or degrading statements regarding clients, customers, or consumers, work colleagues, or other personnel?	• Have you ever made demeaning or degrading statements regarding clients, customers, or consumers? • Have you ever made demeaning or degrading statements about work colleagues? • Have you ever made demeaning or degrading statements about other work personnel? • Have you ever criticized someone from your work?
18	Deterioration in clothing and dressing habits	No	No	Has there been deterioration in your clothing and dressing habits?	Has there ever been deterioration in your clothing and dressing habits?
19	Disorganized schedule	No	Yes	Have you had a disorganized schedule at work?	Has your work schedule ever been disorganized?
20	Intoxicated at social events or odor of alcohol on breath while on duty	No	No	Have you been intoxicated at social events or had odor of alcohol on breath while on duty for work?	• Have you ever been intoxicated at social events? • Have you ever had alcohol on your breath while on work duty?
21	Avoidant, unreliable	No	Yes	Have you been avoidant or unreliable at work?	• Have you ever been avoidant at work? • Have you ever been unreliable at work?
22	Public intoxication or impairment	No	No	Have you been intoxicated or impaired at work?	• Have you ever been intoxicated in public? • Have you ever been impaired in public?
23	Impaired or decreased work performance	No	Yes	Have you had impaired or decreased work performance?	• Has your work performance ever been impaired? • Have you ever had decreased work performance?
24	Smell of alcohol on breath or in perspiration	No	No	Have you had the smell of alcohol on your breath or in perspiration?	• Have you ever had the smell of alcohol on your breath? • Has your perspiration ever smelled of alcohol?
25	Low or elevated self-esteem	No	No	Have you had low or elevated self-esteem?	• Have you ever had low self-esteem? • Have you ever had elevated self-esteem?

*DWI* driving while intoxicated, *DUI* driving under the influence

The complete 571 PHP website descriptions of impairment from 23 states (including the District of Columbia) are available in Additional file 1, which also provides weblinks to these lists, the lists’ titles, the descriptions, and other contextual details. The average number of descriptions provided in a typical PHP list was 24.8 (standard deviation [SD] = 17.8). We arranged lists alphabetically by state and used random number generator on Microsoft Excel to pick a number from 1 to 571 in order to randomly select 25 of these descriptions for inclusion in the survey. We eliminated eight descriptions selected from random number generator because they applied only to physicians or select health care workers (e.g., “Going back into the pharmacy after hours”) and could not be translated into a question for all employees in the general population. These were replaced with the first eight other descriptions that resulted from additional random number generator results.

Wording in the descriptions and additional context on the PHP website specified current time frame in only three of the 25 descriptions. For the narrowly worded version of the survey, we constructed items out of these three descriptions using present tense, and used present perfect tense (e.g., “Have you had….”) for the rest. For the broadly worded version, we used present perfect tense, but added the word “ever” for all of the survey items (e.g., “Have you ever had….”).

Wording and context clearly specified application to the workplace, rather than other settings, in seven of the PHP descriptions, and survey items constructed from these descriptions in both versions made clear that they applied only to work. Another PHP description (“If the resident is experiencing problems coping with patients or with the typical stress of a busy residency”) did not make clear whether the “problems” affected work performance or caused “problems” only in other life contexts, and we preserved that ambiguity in both versions of the survey. Another (“Makes degrading or demeaning comments regarding patients, families, nurses, physicians, hospital personnel, or the hospital….”) did not specify whether the comments were made at work or, for example, at home during discussion with spouse or family, and we did not provide additional specification in either survey version.

Fifteen of the 25 PHP descriptions contained more than one component (e.g., “Intoxicated at social events or odor of alcohol on breath while on duty”), and we broke these into separate questions for the broadly worded version. The survey also contained an additional attention check question, “Can you read this?” Respondents answering “No” to this and all other survey items were eliminated from the final results and analysis.

## Results

**Fig. 1:**
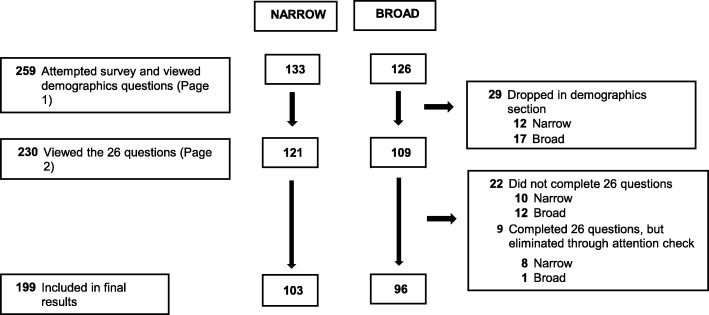
Flow of respondents randomly assigned to either the narrowly worded or broadly worded survey version

Figure 1 displays the flow of respondents throughout the survey. Of the 230 respondents who viewed the 26 survey items (the 25 items listed in Table 1 plus the attention check), 86.5% (199/230) completed the survey and were included in the final results. Table 2 summarizes sociodemographic characteristics of the total sample of 199 respondents. Table 3 gives the percentage of respondents in both survey versions who endorsed each description of impairment. It also lists the mean total number of descriptions endorsed for each survey version.

**Table 2:** Summary data of sociodemographic characteristics of respondents

Characteristic	[mean (SD)] or % (n)
Age in yr	[37.6 (10.6)]
Sex	
Male	47.7 (95)
Race/Ethnicity	
White	77.9 (155)
Hispanic	5.0 (10)
Non-Hispanic black	7.5 (15)
Other	9.5 (19)
Education	
Postgraduate degree	24.1 (48)
Bachelor’s degree	52.8 (105)
High school diploma	23.1 (46)
8th grade	0.0 (0)
Employment	
Full-time	100 (199)

**Table 3:** Endorsement of 25 state physician health program descriptions of impairment in an online sample of respondents currently employed full-time

		% Endorsing description	
	Description	Narrow (*n* = 103)	Broad (*n* = 96)
1	Intimidated someone at work	9.7	19.8
2	Deterioration in personal hygiene	9.7	24.0
3	Constant sadness or tearfulness	14.6	41.7
4	Occurrence of spouse, child abuse	3.9	12.5
5	Easily agitated, irritable	42.7	86.5
6	Increased complaints from customers or clients	4.9	13.5
7	Personality and behavioral changes	19.4	36.5
8	Neglected social commitments	30.1	47.9
9	DWI arrest or DUI violations	4.9	8.3
10	Other mental health concerns that directly impact work performance	17.5	24.0
11	Direct statements indicating distress	24.3	35.4
12	Experiencing problems coping with customers or clients or with typical stress of a busy job	21.4	62.5
13	Sweating when otherwise comfortable	20.4	41.7
14	Tremors, hands shake	17.5	26.0
15	Rapid speech	15.5	35.4
16	Personnel question competence and/or behavior	12.6	26.0
17	Makes degrading or demeaning comments at work	15.5	66.7
18	Deterioration in clothing and dressing habits	13.6	47.9
19	Disorganized work schedule	23.3	22.9
20	Intoxicated at social events or odor of alcohol on breath while on duty	15.5	50.0
21	Avoidant, unreliable	13.6	42.7
22	Public intoxication or impairment	10.7	53.1
23	Impaired or decreased work performance	18.4	43.8
24	Smell of alcohol on breath or in perspiration	13.6	64.6
25	Low or elevated self-esteem	37.9	81.3
Mean (SD) total number of impairment descriptions endorsed		4.3 (5.0)	9.7 (5.8)

*DWI* driving while intoxicated, *DUI* driving under the influence

In the narrow version of the survey, 73 (70.9%) of the 103 respondents endorsed at least one description of impairment, and 61 (59.2%) endorsed more than one. The number of descriptions endorsed for each respondent ranged from 0 to 18 (mean = 4.3, SD = 5.0; median = 2). In the broadly worded version, 93 of 96 (96.9%) respondents endorsed at least one description, and 92 of 96 (95.8%) endorsed more than one. The number of descriptions endorsed ranged from 0 to 21 (mean = 9.7, SD = 5.8; median = 10).

The most commonly endorsed descriptions were “easily agitated, irritable” (42.7% and 86.5% of respondents to narrow and broad versions of the survey, respectively) and “low or elevated self-esteem” (37.9% and 81.3%). The least commonly endorsed were “occurrence of spouse, child abuse” (3.9% and 12.5%) and “DWI arrest or DUI violations” (4.9% and 8.3%).

## Discussion

The results of this study suggest that most of the general US population in full-time employment endorse multiple descriptions of impairment on a typical PHP list. The results also suggest that over 95% of the public who are employed full-time endorse multiple descriptions of impairment when broadly presented, with a median of 10 descriptions out of a typical 25-item list. This means that a typical working member of the public will probably endorse approximately 40% (10/25) of these possible signs and symptoms of “impairment,” and that therefore almost everyone could potentially be regarded as impaired by these descriptions.

Does this mean that employees all over the US are impaired, unable to complete the essential functions of their jobs, or pose a high risk of substantial imminent harm to self or others in the workplace? Of course not. But findings similar to those of this study have been interpreted in this fashion at various points in the past. The National Comorbidity Survey Replication, for example, considered a contemporary gold-standard source on prevalence, found that 44% of the US population aged 18–29 and 37% of those 30–44 meet criteria for a common mental disorder every year [23], and prevalence estimates for members of the medical profession also appear to be at least those of general population [24–26]. Even if it is true that 40% of physicians meet diagnostic criteria for a mental disorder every year, it simply cannot be true that 40% of physicians are impaired. Despite this obvious fact, the authors of these studies call for even *more* increased vigilance, identification, and treatments of physicians with suspected mental disorders [24–26].

The prevalence findings in this paper should not be interpreted as evidence in support of increased identification and evaluations of physician-employees. Our findings that PHP-described impairments are exceedingly broad call into question these descriptions’ ability to identify clinicians who present a meaningful risk of harm. And they should also shine light on the considerable power that these descriptions may provide hospital managers, PHPs, and institutional leaders, who may designate almost anyone as impaired.

The consequences of such mislabeling should be considered in the context of further information regarding physicians’ recourse in the event they are accused of being impaired.

### Recourse for physicians alleged to be impaired

Physicians who are suspected of being impaired and instructed to undergo evaluations often have few avenues of appeal. Residents, for example, may not be able to defend themselves against accusations of impairment. We have seen firsthand how hospital administrators generally side with residency directors, so hospital administrations cannot be considered avenues of appeal. The Accreditation Council for Graduate Medical Education (ACGME) offers no protection for those in training:

[t]he ACGME does not adjudicate disputes between individual persons and residency or fellowship programs or sponsoring institutions regarding matters of admission, appointment, contract, credit, discrimination, promotion, or dismissal of faculty members, residents, or fellows [27].

Residents, who cannot practice medicine without completing a residency program, may not have the resources to hire a lawyer to protect their careers and are almost never successful in court when appealing dismissals [28–30].

Unfair or sham peer reviews of physicians alleged to be impaired have been described in many journals [31–38] but have never been studied systematically. A complaint often raised by physicians accused of being impaired is that their attempts to defend themselves or to oppose referrals to PHPs are often misrepresented as denial or lack of insight, making it harder for them to actually defend themselves [35, 39, 40]. Some leaders of PHPs, however, dispute these complaints [35] as reflective of “a small dissatisfied minority of physicians who are not able to achieve a successful return to their profession.”

Such characterizations, of course, may or may not be accurate. But it is important to bear in mind that PHPs may only be aware of how those actually presenting to PHPs are being affected by their descriptions and outreach efforts. Yet these descriptions may adversely impact many other physicians who are not actually evaluated or treated at a PHP, such as those referred to other providers, and those who simply refuse to comply [5].

### The roles of key groups

Representatives of PHPs have argued that disseminating these descriptions of reasons to refer physicians to their programs is in the best interests of the physicians themselves. This assumes, of course, that these workplace screening procedures result in more, rather than less physicians entering treatment; that these treatments are effective; that any exacerbations of stigma resulting from these screening procedures have, at worst, negligible health effects; and that employers should be involved in monitoring and treating their employees’ health conditions [41]. The descriptions may also be misinterpreted by employers as reasons to remove suspected physicians from practice, request protected health information from them, or refer them for evaluations to PHPs. Such requests, if unwarranted, are illegal under the general rules and regulations of the Americans with Disabilities Act (ADA) [42].

Even though members of state medical societies, PHPs, and medical boards are likely not to be the employers of physicians who might be affected by their pronouncements, they should make every effort to ensure that these descriptions of impairment will not be applied inappropriately within the employment context. Despite this, there appears to have been almost no progress over the last 10 years in removing discriminatory questions from state medical licensure applications [14, 43], nor, as far as we know, any efforts to amend policies on physician impairment within the profession in order to align with the ADA, more than 27 years since it first became law.

Interrelationships among key influential groups may have something to do with the inaction on policy related to physician impairment. The AMA’s policies, for example, are created by the members of its House of Delegates, most of whom are representatives of state medical societies [44], which generally work closely with PHPs and are sometimes (as is the case in Massachusetts) their parent organization. The AMA’s policies on physician impairment were what gave rise to corresponding state laws and medical board regulations on physician impairment [45] and mandate referrals to PHPs.

Through the AMA, state medical societies have called for more research on PHPs by state medical societies [1, 2]. To date, we do not believe the conclusions reached about PHP effectiveness in the medical literature can be fully trusted, since the overwhelming majority of research on PHP outcomes has been performed by representatives of PHPs and/or the evaluation and treatment centers where PHPs often mandate physicians seek treatment [46], and which often have significant conflicts of interest with PHPs [36, 47]. PHP research usually touts very high levels of effectiveness but generally fails to disclose potential bias by authors as well as the fact that key flaws in their study designs limit the conclusions that can reasonably be drawn from these studies [46].

At present, we do not have data about the number of physicians being mislabeled as impaired or inappropriately referred to PHPs. Some journals have published anecdotal accounts of physicians inappropriately labeled as impaired, but to our knowledge, scientific data do not exist. Despite this, we have seen dozens of instances in which physicians who have shown no signs of impairment in the workplace were mandated to undergo evaluations or monitoring with PHPs, or other providers.

We have not seen any PHP (or other professional medical group mentioned previously) post ADA rules on their websites or incorporate ADA rules into their policies. We believe that physicians ought to be aware of their employment rights under the ADA but wonder if these omissions are intentional, given that businesses and occupational health organizations have aggressively lobbied to prevent the US Equal Employment Opportunity Commission (US EEOC) from implementing additional regulations pertaining to workplace wellness programs that could reduce the incidence of prohibited medical inquiries and workplace disability discrimination [48, 49].

### Future directions

Critics of these policies and practices have called for audits of PHPs [40, 47]; antitrust policy [50]; policies for a means of appeal of state medical board/PHP decisions [40, 47]; and revocation of the Health Care Quality Improvement Act (HCQIA) of 1986 and the Patient Safety and Quality Improvement Act of 2005 [31, 34, 35], which reduced physicians’ ability to defend themselves against a hospital’s peer review even more than the HCQIA. Another suggestion has been that state medical boards be replaced by a single, federal entity overseeing these issues [50, 51]. These are all good ideas, but they might not be sufficient to address the problems discussed in this article. The persons made in charge of the federal entity, for example, could end up being be the same people in charge of state medical societies/medical boards/PHPs right now. Calls for these reforms have also so far not been successful.

In our view, these problems call for the involvement of entities working well outside the healthcare professions, as health lawyers may not be aware of the ADA’s rules or may significantly downplay ADA regulations while trumpeting the guidelines provided by professional medical organizations [52]. The reality is that these are disability rights issues. Framing them as healthcare regulatory problems also seems less likely to stimulate reform than framing them as problems of employment discrimination against people with disabilities.

We recommend that the US EEOC adopt strict regulatory standards pertaining to workplace wellness programs in order to reduce the incidence of unwarranted medical inquiries and disability discrimination in the workplace. We also advise all professional entities to help remind members of the medical community of the ADA’s rules, which protect all employees from unwarranted medical inquiries without objective evidence that the employee either:

1. is unable to perform essential job functions due to a medical condition; or
2. poses a high risk of substantial, imminent harm due to a medical condition [42].

While there is some variability between states in mandatory reporting requirements and due process procedures (e.g., standards of proof) for physicians regarded as impaired [53], none can truly be considered reasonable if they remain inconsistent with and do not incorporate the rules of the ADA. Finally, we believe litigation against some of these professional organizations under Title III of the ADA may be needed to discourage interference with implementation of ADA rules and regulations.

### Limitations

The limitations of this study include the fact that it was not conducted on physicians, but rather on members of the general population online through MTurk. MTurk workers in full-time employment may differ in some respects from physicians, but there is little reason to believe that physicians would respond to the survey items included here in ways that are substantially different. That the survey was conducted on members of the general population should not detract from the overall findings that the descriptions are very broad and susceptible to misapplication.

It should also be noted that while some PHPs have assigned titles to their lists of PHP descriptions (e.g., “Signs and Symptoms of Impairment,” “Indicators of Impairment,” and “When to Refer to Professionals Resource Network”) that unequivocally designate the descriptions as indicative of impairment, most provide titles that are somewhat less overt (e.g., “Identification of the Impaired Physician,” “Possible Reasons for Referrals,” “Possible Signs of Impairment”) (see Additional file 1). Nevertheless, these lists are highly suggestive. Though designating these descriptions as “possible” indicators of impairment may seem less objectionable, their repeated suggestion that members of one specific minority group “may” be dangerous or unsuitable for the hospital workplace is problematic. Almost anything can potentially impair one’s performance, but speculative risks are not allowed under the ADA to form the basis of decisions to conduct medical inquiries of employees or refer employees for evaluations [42].

We also have no doubt that some physicians who present voluntarily to PHPs may derive benefit from the services that they receive and have known some physicians who were very grateful toward their work with a PHP. But physician impairment policies, practices, and these descriptions can be nonetheless problematic in their consideration of disabilities and the actions they will take as a result.

## Conclusions

Our findings suggest that PHP descriptions of possible signs of physician impairment are very broad and may be substantially overapplied, leaving almost any physician-employee susceptible to potentially being branded as impaired. Even though the medical leaders responsible for these descriptions and associated policy might not be employers, they may still contribute to unjust work cultures and employer behaviors that discriminate against physician-employees. And to make matters worse, few physicians and health lawyers are actually aware of the provisions of the ADA, which protect all employees from unwarranted medical inquiries and evaluations [42] and supersede conflicting state laws and policies on physician impairment.

To create just cultures, as well as to possibly increase physician engagement in health resources [43, 54], reduce physician suicide [55], medical errors [4], and facilitate inclusion of qualified physicians with these conditions [5], who are not dangerous or inferior clinicians, into the workplace, AMA leaders, state medical societies, medical boards, PHPs, and other professional groups should cease disseminating and promoting these descriptions. They should amend their policies to incorporate the rules of the ADA with regard to prohibited medical inquiries and should not promote other guidance interfering with ADA rules and regulations.

## Additional files

Additional file 1: Physician health program descriptions, weblinks, and additional context. (DOCX 101 kb)

Additional file 2: Checklist for Reporting Results of Internet E-Surveys (CHERRIES). (DOCX 23 kb)

## Abbreviations

ACGME: Accreditation Council for Graduate Medical Education
ADA: Americans with Disabilities Act
AMA: American Medical Association
CHERRIES: Checklist for Reporting Results of Internet E-Surveys
DUI: Driving under the influence
DWI: Driving while intoxicated
HIT: Human intelligence task
MTurk: Amazon’s Mechanical Turk
PHP: Physician health program
SD: Standard deviation
SUD: Substance use disorder
US EEOC: US Equal Employment Opportunity Commission
