# Supplementary material for: How broad are state physician health program descriptions of physician impairment?
Source: Subst Abuse Treat Prev Policy. 2018 Aug 23;13:30. doi: 10.1186/s13011-018-0168-z (PMC6107949; doi:10.1186/s13011-018-0168-z)
Supplement: Supplementary file 1 — Physician health program descriptions, weblinks, and additional context. (DOCX 101 kb) [file 13011_2018_168_MOESM1_ESM.docx]

|  | **Colorado**^a^ 1-58 |
| --- | --- |
|  |  |
|  |  |
| **No.** | **Description** |
|  | **Behavioral Indicators of a Possible Health Problem in Medical Professionals**^b^ |
|  | Family: |
| 1. | Withdrawal from family activities |
| 2. | Children neglected, abused or in trouble |
| 3. | Mood swings, arguments or violent outbursts |
| 4. | Sexual problems; impotence, extramarital affairs |
| 5. | Medicinal use of alcohol or drugs |
| 6. | Family isolation |
| 7. | Financial problems |
| 8. | Spouse in therapy or taking psychoactive medication |
| 9. | Geographical separation or divorce by spouse |
|  | Employment: |
| 10. | Frequent job changes or relocations |
| 11. | Unusual medical history |
| 12. | Indefinite, vague or inappropriate references |
| 13. | Working in positions inappropriate for qualifications |
| 14. | Resistance to pre-employment physical or family interview |
|  | Office: |
| 15. | Disruption in appointment schedule |
| 16. | Hostile, suspicious or unreasonable behavior to staff or patients |
| 17. | Withdrawn, “locked door syndrome” |
| 18. | Excessive ordering of drug supplies |
| 19. | Excessive prescribing practices |
| 20. | Complaints from patients and staff |
| 21. | Unexplained absences from the office |
| 22. | Spasmodic work pace, or decreasing work load and tolerance |
| 23. | Taking sexual advantage of patients or coworkers |
| 24. | Procrastination or neglect of details |
| 25. | Avoidance of fellow workers |
| 26. | Errors in judgment |
| 27. | Becoming a topic of “gossip” |
|  | Legal/Regulatory |
| 28. | Medical licensing issues |
| 29. | Involvement in malpractice suits |
| 30. | Peer review of work |
| 31. | Arrests for driving while intoxicated or other legal problems (i.e. domestic violence, public sexuality acting out) |
|  | Physical |
| 32. | Deterioration in personal hygiene |
| 33. | Deterioration in clothing and dressing habits |
| 34. | Inappropriate dress |
| 35. | Numerous prescriptions and OTC drug use |
| 36. | Frequent ER visits or hospitalizations |
| 37. | Frequent visits to physicians |
| 38. | Accidents |
| 39. | Multiple somatic complaints |
| 40. | Excessive tiredness or insomnia |
| 41. | Memory problems, difficulty concentrating |
| 42. | Emotional crisis |
|  | Community |
| 43. | Neglected social commitments |
| 44. | Inappropriate behavior at social functions |
| 45. | Arrests for driving while intoxicated or legal problems |
| 46. | Unreliability or unpredictability in community activities |
| 47. | Public intoxication or impairment |
|  | Hospital |
| 48. | Making rounds late, or inappropriate or abnormal behavior during rounds |
| 49. | Decreasing quality of performance |
| 50. | Inappropriate orders |
| 51. | Reports of behavioral changes |
| 52. | Unavailability or inappropriate responses to telephone calls |
| 53. | Heavy drinking at staff functions |
| 54. | Often late, absent or ill |
| 55. | Alcohol on the breath when on duty |
| 56. | Intoxicated when on call, even at home |
| 57. | Unreasonable sensitivity to normal criticism from peers |
| 58. | No longer attending committee meetings and/or other functions |
|  |  |

^a^Derived from Colorado Physician Health Program. <http://cphp.org/wp-content/uploads/2016/02/BEHAVIORAL-INDICATORS.pdf> Accessed April 2, 2017.

^a^The same program website page provided additional qualifying “information to consider: The information in this list is an *example* of behavioral indicators of possible health problems in medical professionals and does not serve as a diagnosis of any health condition. This list does not replace a health assessment by a qualified professional. A goal of [the program] is to help physicians before they become impaired. Most ill physicians do not show evidence of impairment at work. Impairment is typically a sign of late stage illness. The most significant behavioral indicator of a health problem is ***a change in mood or attitude***.”

|  | **District of Columbia**^a^ 59-64 |
| --- | --- |
|  |  |
|  |  |
| **No.** | **Description** |
|  | **A Caring Approach Based on the Medical Profession’s Tradition of Healing**^b^ |
|  | Have you ever: |
| 59. | Noticed while at work that a physician colleague smelled of alcohol? |
| 60. | Been concerned by a physician who was so upset and angry with colleagues, nurses or staff that it interfered with patient care? |
| 61. | Been plagued with worry or concern because a colleague just doesn’t seem right? |
|  | Do you know where to turn if: |
| 62. | You thought a physician friend might have a drinking problem? |
| 63. | A colleague was self-prescribing pain-killers or other controlled medications? |
| 64. | A colleague seemed depressed, was experiencing mood instability, or was overly anxious to the point that their performance was being affected? |
|  |  |

^a^Derived from Medical Society of the District of Columbia. <http://c.ymcdn.com/sites/www.msdc.org/resource/resmgr/Docs/phc_brochure_online.pdf> Accessed April 2, 2017.

^b^The text immediately following this information added, “These are some examples of the types of problems that may interfere with the safe practice of medicine or the effective operation of your practice or institution, and have the potential to result in legal or disciplinary actions, which may even affect licensure status. Most importantly, these types of conditions can be addressed through proper assessment and treatment.”

|  | **Florida**^a^ 65-78 |
| --- | --- |
|  |  |
|  |  |
| **No.** | **Description** |
|  | **When to Refer to Professionals Resource Network**^b^ |
| 65. | You suspect alcohol/drugs and/or psychiatric illness may be involved in a workplace incident |
| 66. | Inaccessibility to patients and staff |
| 67. | Decreased workload or workload intolerance and performance |
| 68. | Frequent trips to the bathroom or other unexplained absences |
| 69. | Frequent absences or illness |
| 70. | Sporadic punctuality |
| 71. | Defensive when questioned or confronted |
| 72. | Alcohol on breath with attempts to cover with mints or mouthwash |
| 73. | Deadlines not met |
| 74. | Questionable practice judgment |
| 75. | Unsatisfactory documentation performances |
| 76. | Deteriorating personal hygiene |
| 77. | Personality and behavior changes |
| 78. | Shakiness, tremors of hands, agitation |
|  |  |

^a^Derived from Professionals Resource Network. Referral. <http://www.flprn.org/referral> Accessed April 2, 2017.

^b^The text immediately preceding this information stated, “If you are concerned about yourself or someone else, do not wait until there is a program that may threaten their career, licensure or their life.”

|  | **Georgia**^a^ 79-101 |
| --- | --- |
|  |  |
|  |  |
| **No.** | **Description** |
|  | **Identification of the Impaired Physician**^b^ |
| 79. | High-risk conditions for addiction |
| 80. | Family history of addiction in first-degree relatives |
| 81. | Access to mood-altering medications, particularly opioids, particularly in anesthesiology |
| 82. | Domestic breakdown |
| 83. | Unusual stresses in work |
| 84. | Behaviors of addiction |
| 85. | Use of large quantities of alcohol; frequent drunkenness |
| 86. | Frequent medical complaints without specific diagnoses evident (fatigue, insomnia, indigestion, depression) |
| 87. | Self-prescribing of sedative-hypnotic, opioid medications |
| 88. | Neglect of responsibilities (missing appointments, late to rounds) |
| 89. | Frequent outbursts of anger |
| 90. | Staff concerns about a colleague's behavior |
| 91. | Sexual promiscuity |
| 92. | Driving under the influence citations |
| 93. | Signs of addiction |
| 94. | Smell of alcohol on breath |
| 95. | Ataxic gait |
| 96. | Slurred speech |
| 97. | Unexplained tremor |
| 98. | Disheveled appearance |
| 99. | Somnolence |
| 100. | Unexplained weight changes |
| 101. | Depressed mood |
|  |  |

^a^Derived from Georgia Professionals Health Program. Physicians and substance abuse. <https://gaphp.org/index.php/news/articles/35-physician-impairment-by-substance-abuse> Accessed April 2, 2017.

^b^Data from an academic journal article that was cited and reproduced in full on the program website.

|  | **Idaho**^a^ 102-115 |
| --- | --- |
|  |  |
|  |  |
| **No.** | **Description** |
|  | **Warning Signs** |
| 102. | Excessive drinking |
| 103. | Prescription drug abuse |
| 104. | Finances in turmoil |
| 105. | In need of family counseling |
| 106. | Struggling with addiction |
| 107. | Marital issues |
| 108. | Depression |
| 109. | Erratic behavior |
| 110. | Loss of interest in job |
| 111. | Suicide attempt |
| 112. | Alienation |
| 113. | Changes in weight/appetite |
| 114. | Feelings of hopelessness |
| 115. | Unable to sleep |
|  |  |

^a^Derived from Safe Call Now. Safe Call Now brochure. <https://www.safecallnow.org/brochure---faq-s.html> Accessed April 2, 2017.

|  | **Indiana**^a^ 116-136 |
| --- | --- |
|  |  |
|  |  |
| **No.** | **Description** |
|  | **Common Warning Signs**^b^ |
|  | Office |
| 116. | Disruption of appointment schedule |
| 117. | Hostile, withdrawn or unreasonable behavior toward patients and staff |
| 118. | Excessive ordering of prescription drugs by mail or from local pharmacies |
| 119. | Patient complaints regarding physician’s behavior |
| 120. | Unexplained absence from office or absence due to frequent illnesses |
|  | Hospital |
| 121. | Making rounds late or inappropriately or odd behavior during rounds |
| 122. | Decrease in quality of performance (e.g., incomplete charts) |
| 123. | Increase in number of quality assurance errors |
| 124. | Reports of behavioral changes from hospital personnel |
| 125. | Reports from emergency room staff of unavailability or inappropriate response to telephone calls |
| 126. | Attending emergency patients while appearing under the influence |
| 127. | Frequently late for surgery and meetings |
| 128. | Reports of incoherent phone orders, slurred speech, etc. |
| 129. | Deterioration in appearance and dress habits |
|  | Community |
| 130. | Isolated and withdrawn from community activities, leisure activities or church |
| 131. | Embarrassing behavior at clubs or parties |
| 132. | Unpredictable behavior (e.g., excessive spending, gambling) |
| 133. | Arrests for driving while intoxicated, disorderly conduct |
|  | Signs of Disruptive Behavior |
|  | Some possible signs of disruptive behavior include: |
| 134. | Inappropriate anger or resentments (e.g., intimidation, abusive language, blaming or shaming others, or threats of violence or retribution) |
| 135. | Inappropriate words or actions directed at others (e.g., sexual comments or harassment, seductive or aggressive behavior or ethnic slurs) |
| 136. | Inappropriate responses to patient or staff needs (e.g., chronic late responses to pages or calls, unprofessional conduct, defiance as an approach to problems) |
|  |  |

^a^Derived from Indiana State Medical Association. Physician Assistance Program brochure. <http://www.ismanet.org/pdf/resources/PhysAssistance2008-FINAL.pdf> Accessed April 2, 2017.

^b^A subsection entitled “How to Refer” immediately preceded “Common Warning Sign.” The later described “common signs of distress at the office and hospital. . . .”

|  | **Louisiana**^a^ 137-199 |
| --- | --- |
|  |  |
|  |  |
| **No.** | **Description** |
|  | **Warning Signs**^b^ |
|  | Symptoms of Substance Abuse/Chemical Dependency: |
| 137. | Smell of alcohol on breath or in perspiration |
| 138. | Red-faced and/or prominent capillaries over cheeks and nose |
| 139. | Bloodshot and/or glassy eyes, excessive use of eye drops to conceal it, wears sunglasses indoors |
| 140. | Constricted or dilated pupils |
| 141. | Sweating when otherwise comfortable |
| 142. | Self-medicating, has medical problems and does not seek help, but rather treats self |
| 143. | Memory lapses, can’t account for whereabouts |
| 144. | Slow, slurred, or pressured speech |
| 145. | Avoids close contact or interaction with others, avoids eye contact |
| 146. | Lying |
| 147. | Erratic behavior patterns, mood swings, inappropriate affect (laughing when others are crying) |
| 148. | Tremors, hands shake |
| 149. | Does not answer when on call or does not return pages |
| 150. | Misses work frequently or is late frequently |
| 151. | Defensive and/or minimizing regarding alcohol and/or drug consumption, denial |
| 152. | Persistent financial, marital, or familial problems |
| 153. | History of alcohol or substance abuse in family |
| 154. | Excessive use of fragrance and/or breath spray to conceal substance use |
| 155. | Possession of alcohol or drugs at work (in desk, purse, locker) |
| 156. | Alcohol in car or empty alcohol containers in car on a regular basis |
| 157. | Known to frequent bars/clubs on a regular basis |
| 158. | Frequently associates with known alcohol or substance users/abusers |
| 159. | Low or elevated self-esteem |
| 160. | Poor impulse control, hasty, impatient |
| 161. | Easily agitated, irritable |
|  | Psychiatric Conditions: |
| 162. | Self-medicating, has medical problems and does not seek help, but rather treats self |
| 163. | Erratic behavior patterns, mood swings, inappropriate affect (laughing when others are crying) |
| 164. | Low or elevated self-esteem |
| 165. | Poor impulse control, hasty, impatient |
| 166. | Easily agitated, irritable |
| 167. | Psychosomatic, hypochondriasis |
| 168. | Paranoia |
| 169. | Insomnia, hypersomnia |
| 170. | Anxious |
| 171. | Depressed, flat affect |
| 172. | Manic affect |
| 173. | Distorted thinking, delusional, hallucinations, disconnected thoughts |
| 174. | Isolative, withdrawn |
| 175. | Denial, minimizes having problems |
| 176. | Suicidal ideation or previous attempt |
| 177. | Pattern of extreme risk-taking behavior |
| 178. | Misses work frequently or is late frequently |
| 179. | Reputation of bizarre or otherwise extreme behavior |
| 180. | Passive-aggressive and/or manipulative behavior |
|  | Disruptive Behavior: |
| 181. | Abrasive interpersonal style of interaction |
| 182. | Passive-aggressive and/or manipulative behavior |
| 183. | Appearance of never being happy or satisfied |
| 184. | Apathetic, pessimistic, believes things will never improve |
| 185. | Bizarre, unusual behavior |
| 186. | Often grandiose, elevated self-esteem |
| 187. | Is threatening, verbally abusive |
| 188. | Often overreacts |
| 189. | Very low tolerance for others, while expecting others to have great tolerance for him/her |
| 190. | Often acts with disregard for rules and consequences, feels entitled to do so |
| 191. | Often complains and is often complained about to authorities for rude or otherwise unacceptable behavior |
| 192. | Denies wrongdoing and refuses to make changes in behavior |
|  | Physical Limitation: |
| 193. | Appears unable to see when others have no difficulty |
| 194. | Weak or restricted grip (hand shake) |
| 195. | Tremors, hands shake, deterioration of fine motor skills |
| 196. | Unsteady gait |
| 197. | Frequent loss of balance, diminished equilibrium |
| 198. | Cannot raise arms above head, bend and touch toes, etc. |
| 199. | Muscle, bone, nerve or tendon damage |
|  |  |

^a^Derived from Health Professionals’ Foundation of Louisiana. Warning signs. <http://hpfla.org/warning-signs> Accessed April 2, 2017.

^a^Additional text, on the same website page as these descriptions, clarified that “[a]ny of these symptoms alone do not constitute impairment, but when taken in combination can be a reliable indicator.  Patterns of behavior are what is important, not single, isolated events or episodes, unless they are so extraordinary that they endanger the professional or others.  Also, it is not uncommon for a professional to simultaneously have more than one impairment type.”

|  | **Maine**^a^ 200-219 |
| --- | --- |
|  |  |
|  |  |
| **No.** | **Description** |
|  | **Signs and Symptoms of Impairment** |
|  | Changes in Work Habits |
| 200. | Conflicts with colleagues |
| 201. | Absenteeism/lateness |
| 202. | Increased patient complaints |
| 203. | Neglect of patients or duties |
| 204. | Appointments/schedules disorganized |
| 205. | Decreased productivity |
| 206. | Misses work or frequently is tardy because of illness or oversleeping |
| 207. | Doesn't keep scheduled appointments |
| 208. | Assignments are late and work is unacceptably inaccurate |
| 209. | Narcotic inventory counts are consistently off |
| 210. | Going back into the pharmacy after hours |
|  | Changes in Behavior |
| 211. | Has become more irritable, defensive, jealous, easily angered, depressed or moody and these behaviors affect work and relationships at work |
| 212. | More withdrawn socially or professionally |
| 213. | Alcohol on breath |
| 214. | Unexplained weight change |
| 215. | Anxiety |
|  | Change in Personal Care |
| 216. | Personal hygiene is deteriorating |
|  | Changes in Prescribing Practices |
| 217. | Writing prescriptions for narcotics, stimulants or sedatives for self or office staff |
| 218. | Requesting prescriptions for narcotics, stimulants or sedatives from colleagues |
| 219. | Diverting patient’s narcotics, stimulants or sedatives for self-use |
|  |  |

^a^Derived from Medical Professionals Health Program. Signs and symptoms of impairment. <https://www.mainemed.com/sites/default/files/content/MPHP%20Brochure%202013.pdf> Accessed April 2, 2017.

|  | **Massachusetts**^a^ 220-244 |
| --- | --- |
|  |  |
|  |  |
| **No.** | **Description** |
|  | **Signs of Concern** |
|  | Personality Changes |
| 220. | Direct statements indicating distress |
| 221. | Constant sadness or tearfulness |
| 222. | Constant anxiety or irritability |
| 223. | Unprovoked anger or hostility |
| 224. | Expressions of hopelessness or worthlessness |
|  | Behavioral Changes |
| 225. | Deterioration in quality of work |
| 226. | A negative change in performance |
| 227. | Repeated absences from important activities |
| 228. | Continual seeking of special accommodations |
| 229. | Repeated trouble getting along with others |
|  | Physical Changes |
| 230. | Deterioration in physical appearance |
| 231. | Visible weight changes |
| 232. | Excessive fatigue |
| 233. | Sleeping much more or much less |
| 234. | Coming to work bleary-eyed or smelling of alcohol |
| 235. | Needle marks or bandages |
|  | Workplace Changes |
| 236. | Patient or staff complaints |
| 237. | Unusual pattern of prescribing of drugs |
| 238. | Inappropriate orders |
| 239. | Personal administration of drugs to patients |
| 240. | Unreachable when on call |
|  | Other Factors to Consider |
| 241. | Personal losses (e.g., divorce, deaths) |
| 242. | Decreased self-care (e.g., discontinuing exercise) |
| 243. | Expression of concern noted by peers |
| 244. | A hunch or gut-level feeling on your part that something is wrong |
|  |  |

^a^Derived from Physician Health Services. Signs of concern. <http://www.massmed.org/Physician_Health_Services/Helping_Yourself_and_Others/Signs_of_Concern/#.WRJWSSyGPIU> Accessed April 2, 2017.

|  | **Michigan**^a^ 245-262 |
| --- | --- |
|  |  |
|  |  |
| **No.** | **Description** |
|  | **Potential Signs of Impairment . . .** |
|  | Emotional or Behavioral Changes: |
| 245. | More withdrawn socially or professionally |
| 246. | More irritable, anxious, jealous, angry, depressed or moody |
| 247. | More defensive - becoming angry when someone mentions their use of drugs, drinking or emotional instability |
| 248. | Denying or expressing guilt or shame about personal use |
| 249. | Other mental health concerns that directly impact work performance |
|  | Change in Work Habits: |
| 250. | Missing work or frequently tardy |
| 251. | Failing to keep scheduled appointments |
| 252. | Late submissions of reports or assignments |
| 253. | Asking others to cover for hours or errors |
| 254. | Unacceptable error rates |
| 255. | Volunteering for drug-oriented tasks |
|  | Physical Changes: |
| 256. | A deterioration in personal hygiene |
| 257. | Changes in eating patterns or body weight |
| 258. | Changes in sleeping patterns |
|  | Substance Use / Addiction |
| 259. | Documented diversion of controlled substances |
| 260. | Observed intoxicated behavior within the workplace |
| 261. | Reports of positive drug screens |
| 262. | Behavior that indicates impairment or addiction |
|  |  |

^a^Derived from Michigan Health Professional Recovery Program. Health Professional Recovery Program brochure. <http://www.hprp.org/documents/HPRP%20Brochure.pdf> Accessed April 2, 2017.

^b^The section began, “The following are common signs of impairment due to substance use or mental health disorders. A health care professional who exhibits several of these common signs may be impaired.”

|  | **Mississippi**^a^ 263-266 |
| --- | --- |
|  |  |
|  |  |
| **No.** | **Description** |
|  | **Possible reasons for referrals** |
| 263. | Boundary violations with staff or patients |
| 264. | Disruptive behaviors (physical or verbal) towards patients or staff |
| 265. | Sexual inappropriateness with patients or staff (violations against patients are immediately reported to the Medical Boards.) |
| 266. | Being under the influence of alcohol or controlled substances in the workplace |
|  |  |

^a^Derived from Mississippi Physician Health Program. Make a referral. <http://msphp.com/make-a-referral> Accessed April 2, 2017.

|  | **Montana**^a^ 267-274 |
| --- | --- |
|  |  |
|  |  |
| **No.** | **Description** |
|  | **What are the Warning Signs?**^b^ |
| 267. | Are you experiencing problems coping with patients or the normal stress of busy practice? |
| 268. | Do you become easily depressed, angered or abusive? |
| 269. | Do you consume more than a moderate amount of alcohol or is your drinking considered "out of control"? |
| 270. | Do you self-prescribe any medications? |
| 271. | Are you experiencing any sexual problems: impotency or affairs? |
| 272. | Do you find yourself slowing down or overtired? |
| 273. | Do you constantly place a precedence for work over personal and/or family needs? |
| 274. | Are you experiencing financial or legal problems: malpractice suit, divorce, DUI? |
|  |  |

^a^Derived from Montana Professional Assistance Program. The process. <http://www.montanaprofessionalassistance.com/process.htm> Accessed April 2, 2017.

^b^The website page stressed the importance of referrals to the program as a consequence of physicians’ resistance to engaging in this treatment voluntarily. This subsection began, “If you can answer yes to one or more of the following questions, you may be in need of our help.”

|  | **North Carolina**^a^ 275-290 |
| --- | --- |
|  |  |
|  |  |
| **No.** | **Description** |
|  | **Common Warning Signs of a Substance Use Disorder** |
|  | Attitude/Behavior Changes |
| 275. | Rapidly turns from compassionate and caring to abrupt and caustic |
| 276. | Withdraws from friends and activities |
| 277. | Becomes mistrusting, anxious, depressed, irritable |
|  | Physical Changes |
| 278. | Loss of appetite or reduced level of exercise |
| 279. | Looks tired; admits to insomnia |
| 280. | Deterioration of personal hygiene |
| 281. | Physical problems are self-treated |
| 282. | Smell of alcohol on breath while on duty |
| 283. | Euphoria or somnolence without cause |
| 284. | Red or glassy eyes |
|  | Performance Changes |
| 285. | Misses appointments |
| 286. | Makes rounds at unusual hours |
| 287. | Cannot be reached when on call |
| 288. | Sloppy charting |
| 289. | Late to work/leaves early |
| 290. | Frequent absences |
|  |  |

^a^Derived from North Carolina Physicians Health Program. Warning signs. <https://ncphp.org/warning-signs/> Accessed April 2, 2017.

|  | **Nevada**^a^ 291-300 |
| --- | --- |
|  |  |
|  |  |
| **No.** | **Description** |
|  | **Top 10 Warning Signs:** |
| 291. | Neglect of family and isolation from friends |
| 292. | Worksite is effected |
| 293. | Personality changes, including irritability, moodiness, argumentative |
| 294. | Absences from work, especially on Monday, attributed to financial, family, or social crisis |
| 295. | Working at unusual hours—late in the evening or very early in the morning |
| 296. | Deficits in quality of work |
| 297. | Professional license at risk |
| 298. | Unexplained accidents or injuries |
| 299. | Intoxicated at social events |
| 300. | Arrested for DUI |
|  |  |

^a^Derived from Clinical Services of Nevada. Nevada Professionals Assistance Program brochure. <http://clinicalservicesnv.com/npap.pdf> Accessed September 7, 2016.

^b^The words, “How Do You Know If You, or A Loved One, Needs Help?” immediately preceded this subsection on “Top 10 Warning Signs.”

|  | **New York**^a^ 301-322 |
| --- | --- |
|  |  |
|  |  |
| **No.** | **Description** |
|  | **Possible Indications of Impairment** |
| 301. | Unkempt appearance, poor hygiene |
| 302. | Trembling, slurred speech |
| 303. | Bloodshot or bleary eyes |
| 304. | Complaints by patients and nurses |
| 305. | Arguments, bizarre behavior |
| 306. | Irritability, depression, mood swings |
| 307. | Irresponsibility, poor memory, poor concentration |
| 308. | Unexplained accidents or injuries to self |
| 309. | Neglect of family, isolation from friends |
| 310. | DWI arrest or DUI violations |
| 311. | Financial and/or legal problems |
| 312. | Difficult to contact; won't answer phone or return calls |
| 313. | Dwindling medical practice |
| 314. | Missed appointments, unexplained absences |
| 315. | Rounds at irregular times |
| 316. | Loss of interest in professional activities, social or community affairs |
| 317. | Neglect of patients, incomplete charting, or neglect of other medical staff duties |
| 318. | Inappropriate treatment or dangerous orders |
| 319. | Excessive prescription writing |
| 320. | Unusually high doses or wastage noted in drug logs |
| 321. | Noticeable dependency on alcohol or drugs to relieve stress |
| 322. | Intoxicated at social events or odor of alcohol on breath while on duty |
|  |  |

^a^Derived from Medical Society of the State of New York. Committee for Physician Health referrals. <http://www.mssny.org/MSSNY/CPH/Referrals/MSSNY/Physician_Advocacy/CPH-Physician_Health/Referrals.aspx?hkey=393fe21c-cfa6-4e75-9b9e-19cc94ad92a7> Accessed April 2, 2017.

|  | **Oklahoma** 323-390 |
| --- | --- |
|  |  |
|  |  |
| **No.** | **Description** |
|  | **Who or What is a Distressed Physician?**^a,b^ |
| 323. | Employs threatening or abusive language, directed at nurses, hospital personnel, or other physicians (e.g. belittling, berating, and/or threatening another individual). |
| 324. | Makes degrading or demeaning comments regarding patients, families, nurses, physicians, hospital personnel, or the hospital. |
| 325. | Uses profanity or other grossly offensive language while in a professional setting. |
| 326. | Utilizes threatening or intimidating physician contact. |
| 327. | Makes public or derogatory comments about the quality of care being provided by other physicians nursing personnel or the hospital. |
| 328. | Writes inappropriate medical records entries concerning the quality of care being provided by the hospital or any other individual. |
| 329. | Imposes idiosyncratic requirements on ancillary staff which have nothing to do with better patient care, but serve only to burden staff with “special” techniques and procedures. |
|  | **Chemical Dependency: Indicators of Impairment** |
|  | Personal |
| 330. | Negative world view characterized by anxiety, depression, guilt, anger |
|  | Multiple medical problems: |
| 331. | hypertension |
| 332. | tachycardia |
| 333. | gout |
| 334. | gastritis |
| 335. | sleep disturbances |
| 336. | sexual problems |
| 337. | Self-medication including mood altering chemicals |
| 338. | Delusional ideation, i.e., “It can’t happen to me.” |
| 339. | Frequent or unusual accidents |
| 340. | DWI charges or other legal difficulties |
| 341. | Behavior that is in conflict with personal values |
|  | Family and Interpersonal |
| 342. | Family conflict |
| 343. | Isolation and withdrawal from family and family activities |
| 344. | Unexplained absences |
| 345. | Acting out behavior by children |
| 346. | Dissolution of marriage |
|  | Increasing dysfunctional family system |
| 347. | Spouse and other family members assume duties and obligations of impaired person |
| 348. | Family attempt to “cover up” for the impaired person’s behavior |
| 349. | Family members accept blame for the person’s impairment |
| 350. | Unsuccessful attempts to “normalize” the family |
| 351. | Family or spouse develop a negative world view characterized by fear, anxiety, depression, guilt and anger |
| 352. | Withdrawal from normal community activities and relationships |
|  | Work History or Resume Clues |
| 353. | Unexplained gaps in resume |
| 354. | History of previous limitations on licensure |
| 355. | Frequent job changes and “geographical cures” |
| 356. | Problem of verifying references or resume itself |
| 357. | Unusual medical problems or disabilities |
| 358. | Application for specific job does not seem compatible to level of professional training |
| 359. | Reluctance to have laboratory tests done or physical exam performed |
| 360. | Isolation from professional community |
| 361. | Insurability problems |
|  | Office |
| 362. | Inappropriate behavior at medical meetings |
| 363. | Concern by Associates of change in attitude and/or behavior |
| 364. | Adverse changes in professional demeanor |
| 365. | Long absences from the office with disruption of patient appointments |
|  | Associates become “professional enablers” |
| 366. | Assume impaired person’s duties |
| 367. | Makes excuses for impaired person |
| 368. | Try to “wait it out” |
| 369. | Become embarrassed by the impaired person’s behavior |
| 370. | Complaints by office personnel and/or patients |
| 371. | Malpractice suites |
| 372. | Complaints to grievance committees |
| 373. | Changes in ordering and prescribing practices regarding mood altering drugs |
|  | Professional Relationships (Hospital) |
| 374. | Avoidance of peers |
| 375. | Making rounds at unusual times |
| 376. | Missing rounds |
| 377. | Patient complaints |
| 378. | Inappropriate dress and/or hygiene |
| 379. | Complaints to grievance committees |
| 380. | Changes in ordering and prescribing practices regarding mood altering drugs |
| 381. | Change in handwriting |
| 382. | Change in quality of progress notes |
| 383. | Unusual or inappropriate orders |
| 384. | Excessive laboratory tests |
| 385. | Excessive consultation |
| 386. | Poor response time to hospital pages, i.e., “The broken beeper syndrome” |
| 387. | Hospital personnel question competence and/or behavior |
| 388. | Frequent citations by review panels, i.e., quality assurance panels |
| 389. | Involvement in litigation against hospital |
| 390. | Practice in areas of medicine for which the person is not qualified |
|  |  |

^a^Derived from Oklahoma Health Professionals Program. Distressed physicians. <https://www.okhpp.org/distressed-physicians-2/> Accessed April 2, 2017.

^b^The section began, “The distressed physician typically exhibits a pattern of behavior characterized by one or more of the following actions,” and later clarified, “that we are talking about a ***pattern of behavior*** that ***may or may not*** overlap a psychiatric diagnosis and/or other impairment such as chemical dependence, major depression, or personality disorder.” The author of the program website also opined, “Typically, the clinician has little or no insight,” and described a strategy for hospitals to develop legal documentation and lay the groundwork for eventual referral to the program.

^c^Derived from Oklahoma Health Professionals Program. Chemical dependency. <https://www.okhpp.org/chemical-dependency/> Accessed April 2, 2017

|  | **Pennsylvania**^a^ 391-408 |
| --- | --- |
|  |  |
|  |  |
| **No.** | **Description** |
|  | **Possible Indications of Impairment** |
|  | Physical Appearance |
| 391. | Wearing long sleeves in warm weather |
| 392. | Deterioration of hygiene or appearance |
| 393. | Frequent or unusual accidents |
|  | Family or Home |
| 394. | Marital or sexual problems |
| 395. | Personality or behavior changes |
| 396. | Medicinal use of alcohol or drugs |
| 397. | Unexplained absences from home |
| 398. | Withdrawal from family activities |
| 399. | Financial problems |
|  | Community |
| 400. | Unreliability or neglecting commitments |
| 401. | Isolation or withdrawal |
| 402. | Unpredictable behavior |
| 403. | Embarrassing behavior at social functions |
| 404. | Arrest for DUI or other legal problems |
|  | Office/Hospital |
| 405. | Frequent or unexplained absences |
| 406. | Excessive working |
| 407. | Inaccessible ("locked door syndrome") |
| 408. | Desire to work alone or refusing work relief |
|  |  |

^a^Derived from Pennsylvania Medical Society. Recognizing impairment. <https://www.pamedsoc.org/foundation/physicians-health-program/recognizing-impairment> Accessed April 2, 2017.

|  | **Rhode Island**^a^ 409-425 |
| --- | --- |
|  |  |
|  |  |
| **No.** | **Description** |
|  | **Common Warning Signs**^b^ |
|  | Attitude/Behavior Changes |
| 409. | Rapidly turns from compassionate and caring to abrupt/caustic |
| 410. | Withdraws from friends and activities |
| 411. | Becomes mistrusting, anxious, depressed, irritable |
|  | Physical Changes |
| 412. | Loss of appetite or reduced level of exercise |
| 413. | Looks tired; admits to insomnia |
| 414. | Personal hygiene deteriorates |
| 415. | Physical problems are self-treated |
|  | Performance Changes |
| 416. | Misses appointments |
| 417. | Makes rounds at unusual hours |
| 418. | Can’t be reached when on call |
| 419. | Sloppy charting |
| 420. | Smell of alcohol on breath during the day |
|  | Relationship Changes |
| 421. | Family communication deteriorates |
| 422. | Frequent arguments, spouse blamed |
| 423. | Occurrence of spouse, child abuse |
| 424. | Children may exhibit poor school performance |
| 425. | Jealousy, infidelity leading to separation, divorce |
|  |  |

^a^Derived from Rhode Island Medical Society. Physician Health Program. <http://www.rimedicalsociety.org/physician-health-program.html> Accessed April 2, 2017.

^b^The website explains that “[t]he presence of the following signs can identify an individual whose health or performance may be impaired. Even if you are not certain a problem exists, it is important to contact the Physician Health Program for assistance.”

|  | **South Dakota** 426-447 |
| --- | --- |
|  |  |
|  |  |
| **No.** | **Description** |
|  | **Signs of Concern of Behavioral Illness for Professionals** |
|  | Personal |
| 426. | Deteriorating personal hygiene |
| 427. | Multiple physical complications |
| 428. | Personality and/or behavioral |
| 429. | Rapid or pressured speech |
| 430. | Mood swings |
| 431. | Bizarre behavior |
| 432. | Inappropriate anger and/or abusive language |
| 433. | D.U.I./Legal problems |
|  | Professional |
| 434. | Disorganized schedule |
| 435. | Erratic behavior- arguments or altercations with patients and/or staff |
| 436. | Inaccessibility to patients and/or staff, patient complaints, calls not being returned |
| 437. | Unable to keep up with the workload |
| 438. | Frequent lateness, absence, or illness |
| 439. | Impaired or decreased work performance |
| 440. | Poor and/or untimely record keeping |
| 441. | Failure to respond to requests to catch up |
| 442. | Inappropriate orders |
| 443. | Disregard of practice standards, institutional rules or laws |
| 444. | Inappropriate response to patient's needs, supervisor, or staff requests |
| 445. | Unprofessional demeanor or conduct |
| 446. | Uncooperative, defiant approach to problems |
| 447. | Disruptive behaviors |
|  |  |

^a^Derived from Midwest Health Management Services. Signs of concern. <http://www.mwhms.com/signs-of-concern.html> Accessed April 2, 2017.

|  | **Tennessee**^a^ 448-473 |
| --- | --- |
|  |  |
|  |  |
| **No.** | **Description** |
|  | **Distressed Conduct**^b^ |
|  | Inappropriate anger or resentment |
| 448. | Inappropriate anger or resentment |
| 449. | Intimidation |
| 450. | Abusive language |
| 451. | Demeaning other staff |
| 452. | Blaming or shaming others for possible adverse outcomes |
| 453. | Unnecessary sarcasm or cynicism |
| 454. | Threats of violence, retribution or litigation |
|  | Inappropriate words or actions directed toward another person |
| 455. | Sexual comments, jokes or innuendo |
| 456. | Flirtation, sexual harassment |
| 457. | Seductive, aggressive or assaultive behavior |
| 458. | Racial, ethnic or socioeconomic bias or slurs |
| 459. | Lack of regard for personal comfort and dignity of others |
|  | Inappropriate responses to patient needs or staff requests |
| 460. | Uncooperative, defiant, rigid, inflexible |
| 461. | Avoidant, unreliable |
| 462. | Late or unsuitable replies to pages and calls or exaggerated response |
| 463. | Unprofessional demeanor or conduct |
| 464. | Arrogant, disrespectful |
| 465. | Inadequate communication in quantity, quality and promptness |
| 466. | Recurrent conflict with others, particularly authority figures; irrational, oppositional |
|  | Some specific examples include: |
| 467. | Employs threatening or abusive language directed at nurses, hospital personnel, or other physicians (e.g. belittling, berating, and/or threatening). These attacks usually are personal, irrelevant, and go beyond the bounds of fair professional comment. |
| 468. | Makes degrading or demeaning comments regarding patients, families, nurses, physicians, hospital personnel, or the hospital. The physician’s non-constructive criticism often works to intimidate, undermine confidence, belittle, or imply stupidity or incompetence in his or her victims. |
| 469. | Uses profanity or other grossly offensive language while in a professional setting. Refuses to accept medical staff assignments or participate in committee or departmental affairs on anything but his or her own terms. |
| 470. | Utilizes threatening or intimidating physical contact. |
| 471. | Makes public derogatory comments about the quality of care being provided by other physicians, nursing personnel, or the hospital. |
| 472. | Writes inappropriate medical records entries concerning the quality of care being provided by the hospital or any other individual. (One may find illustrations in patient medical records, or other official documents. These communications are designed to impugn the quality of care in the hospital or attack particular physicians, nurses.) |
| 473. | Imposes idiosyncratic requirements on ancillary staff which have nothing to do with better patient care, but serve only to burden staff with “special” techniques and procedures. |
|  |  |

^a^Derived from Tennessee Medical Foundation. Handling distressed physician behavior. <https://e-tmf.org/app/uploads/2016/02/TMF-Distressed-Physician-Behavior2016.pdf> Accessed April 2, 2017.

^b^The webpage contained the following text immediately before this list, stating, “Distressed conduct is more than unusual or unorthodox behavior. It typically involves a pattern of behavior characterized by one or more of the following actions.” The webpage also contained the following text immediately after the above list, stating, “Note that we are talking about a pattern of behavior that may or may not overlap a psychiatric diagnosis and/or other impairment such as chemical dependence, major depression or personality disorder. The presence or absence of a diagnosis is important for many reasons, including the ability of the [program] to help. The presence of a pattern is also very important. The [program] usually does not (and generally should not) receive referrals for an isolated incident or very minor instances of distressed behavior.

“A hospital is an especially stressful working environment, so outbursts or other misconduct that probably would not be tolerated elsewhere are often excused. If an isolated outburst is followed by an apology, there is most likely not a longer-term problem. There are clearly limits to tolerance, however. When a physician’s conduct disrupts the operation of the hospital, affects the ability of others to get their jobs done, creates a ‘hostile work environment’ for hospital employees or other physicians on the medical staff, or begins to interfere with the physician’s own ability to practice competently, action must be taken.”

|  | **Texas** 474-525 |
| --- | --- |
|  |  |
|  |  |
| **No.** | **Description** |
|  | **When do residents need help?**^a^ |
| 474. | If the resident is experiencing problems coping  with patients or with the typical stress of a busy  residency; |
| 475. | If the resident becomes easily depressed or  annoyed; |
| 476. | If the resident drinks more than a moderate  amount; |
| 477. | If the resident self-prescribes mood-altering drugs; or |
| 478. | If the resident is slowing down, overly tired, or constantly placing work ahead of personal needs, family, or recreation. |
|  | **Early Signs and Symptoms of Problem Behavior**^b,c^ |
| 479. | Excessive work may be an early retreat from overwhelming personal and professional conflicts. |
| 480. | Working hours become irregular and inefficient. |
| 481. | Sleeping and eating habits become poor and irregular. |
| 482. | The physician may withdraw from social and family activities. |
|  | **Continuing Signs**^d^ |
| 483. | The physician begins to have difficulties in the diagnosis and management of patients. |
| 484. | The physician may be afraid to refer patients because contact with colleagues may expose perceived or actual deficiencies in patient care. |
| 485. | Hospital rounds begin to be made at unusual hours or on a schedule different from colleagues. |
| 486. | The physician is difficult to contact, and nurses may complain of lack of availability. |
|  | Family^e^ |
| 487. | Unexplained absences from home; |
| 488. | Isolates or withdraws from children or spouse; |
| 489. | Children develop behavioral problems; |
| 490. | Sexual dysfunction; and |
| 491. | Separation or divorce. |
|  | Career |
| 492. | Employed in positions not appropriate for training and qualifications; |
| 493. | Increasing malpractice incidents; |
| 494. | Vague letters of reference; and |
| 495. | Unexplained time lapses between jobs. |
|  | Hospital |
| 496. | Unprofessional behavior; e.g., during rounds; |
| 497. | Inappropriate orders; |
| 498. | Quality of charting deteriorates; |
| 499. | Frequently late or absent; |
| 500. | Unavailable for emergency room or call; |
| 501. | Increased patient complaints; |
| 502. | Malpractice suits and legal sanctions; |
| 503. | Atypical times for hospital rounds; and |
| 504. | Deterioration of relationship with staff and patients. |
|  | Office |
| 505. | Deterioration of relationship with staff and patients; |
| 506. | Increased complaints about physician’s behavior; |
| 507. | Frequently late or absent, with appointment schedule disruptions; |
| 508. | Self-prescribes (particularly opiates and/or benzodiazepines); and |
| 509. | Orders excessive amounts of drugs by mail. |
|  | Community |
| 510. | Isolates or withdraws from activities; |
| 511. | Unpredictable personal behavior, including high-risk behaviors; |
| 512. | Heavy drinking or embarrassing behavior at parties; and |
| 513. | Arrests for DWI or other legal problems. |
|  | Behavioral Changes |
| 514. | Multiple accidents or traumatic injuries; |
| 515. | Frequent medical illness and absence; |
| 516. | Prescriptions for self and family; |
| 517. | Self-medicating to change mood; |
| 518. | Personal hygiene and dress deterioration; and |
| 519. | Poor eating and sleeping habits. |
|  | Emotional/Cognitive Changes |
| 520. | Depression; |
| 521. | Mood swings; |
| 522. | Poor concentration; |
| 523. | Confusion; |
| 524. | Sleep disturbance; and |
| 525. | Anxiety/Agitation. |
|  |  |

^a^Derived from Texas Medical Association Committee on Physician Health and Wellness. Do you know a resident who needs our help? [https://www.texmed.org/uploadedFiles/ Current/2016_About_TMA/Physician_Assistance/Do%20You%20Know%20a%20Resident%20Who%20Needs%20Our%20Help.pdf](https://www.texmed.org/uploadedFiles/%20Current/2016_About_TMA/Physician_Assistance/Do%20You%20Know%20a%20Resident%20Who%20Needs%20Our%20Help.pdf) Published April 19, 2002. Updated March 2014. Accessed April 2, 2017.

^b^Derived from Texas Medical Association Committee on Physician Health and Wellness. Substance use disorders among physicians. [https://www.texmed.org/uploadedFiles/Current/2016_About_TMA/ Physician_Assistance/Substance%20Use%20Disorders%20Among%20Physicians.pdf](https://www.texmed.org/uploadedFiles/Current/2016_About_TMA/Physician_Assistance/Substance%20Use%20Disorders%20Among%20Physicians.pdf) Accessed April 2, 2017.

^c^Webpage text immediately beneath this heading read, “Problem behaviors that may be early signs of physician impairment are as follows.”

^d^Webpage text immediately beneath this heading read, “Problem behaviors that may show up in later stages of physician impairment are as follows.”

^e^Webpage text immediately preceding this subheading read, “These are several areas of a physician’s life affected by SUDs.”

|  | **West Virginia**^a^ 526-549 |
| --- | --- |
|  |  |
|  |  |
| **No.** | **Description** |
|  | **Warning Signs** |
| 526. | Absenteeism, especially on Monday |
| 527. | Tardiness |
| 528. | Unavailable while on call |
| 529. | Alcohol on breath |
| 530. | Mood changes |
| 531. | Anxious |
| 532. | Appearing depressed |
| 533. | Deterioration of personal hygiene |
| 534. | Irritability |
| 535. | Hyperactivity |
| 536. | Isolation |
| 537. | Impulsiveness |
| 538. | Inflexible |
| 539. | Irrational behavior |
| 540. | Personality & behavioral changes |
| 541. | Neglect of social commitments |
| 542. | Working long hours |
| 543. | Self-prescribing |
| 544. | Medication diversion |
| 545. | Complaints from other medical staff |
| 546. | Complaints from patients |
| 547. | D.U.I. / legal problems |
| 548. | Frequent job changes or relocation |
| 549. | Deterioration of clinical performance |
|  |  |

^a^Derived from West Virginia Medical Professionals Health Program. Warning signs. <http://www.wvmphp.org/Warning-Signs.html> Accessed April 2, 2017.

|  | **Wyoming**^a^ 550-571 |
| --- | --- |
|  |  |
|  |  |
| **No.** | **Description** |
|  | **C.A.G.E. Questionnaire** |
| 550. | Have you ever felt you should CUT down on your drinking? |
| 551. | Have people ANNOYED you by criticizing your drinking? |
| 552. | Have you felt bad or GUILTY about your drinking? |
| 553. | Have you ever had a drink first thing in the morning to steady your nerves or to get rid of a hangover; EYE opener? |
|  | **Burnout Questionnaire** |
| 554. | Have you become cynical or critical at work? |
| 555. | Do you drag yourself to work and have trouble getting started once you arrive? |
| 556. | Have you become irritable or impatient with co-workers, customers or clients? |
| 557. | Do you lack the energy to be consistently productive? |
| 558. | Do you lack satisfaction from your achievements? |
| 559. | Do you feel disillusioned about your job? |
| 560. | Are you using food, drugs or alcohol to feel better or to simply not feel? |
| 561. | Have your sleep habits or appetite changed? |
|  | **Signs of Not Coping Well:**^b^ |
| 562. | Changes in sleep habits; too much, too little |
| 563. | Changes in eating habits |
| 564. | Unable to shake off feeling down or blue |
| 565. | Increased use of alcohol or other drugs |
| 566. | Feeling like “life is just not worth it” |
| 567. | Feeling like “everything is hopeless” |
| 568. | Difficulty concentrating, distracted, in a fog |
| 569. | Unable to control anger, irritable over little things |
| 570. | Crying more or shutting down feelings; isolating |
| 571. | Fighting with family, friends, colleagues |
|  |  |

^a^Derived from Wyoming Professional Assistance Program. Self tests. <http://www.wpapro.org/self-tests.html> Accessed April 2, 2017.

^b^Beneath the list of “Signs of Not Coping Well,” the website reads, “If you or a colleague is experiencing any of the above, [we] can help.”

DUI: driving under the influence; DWI: driving while intoxicated; ER: emergency room; OTC: over-the-counter

Gray shading indicates criteria that were randomly selected and included in the current study, while red shading indicates criteria that were randomly selected but were not included because of features that rendered them not applicable to the general US population in full-time employment.
