# Supplementary material for: How broad are state physician health program descriptions of physician impairment?
Source: Subst Abuse Treat Prev Policy. 2018 Aug 23;13:30. doi: 10.1186/s13011-018-0168-z (PMC6107949; doi:10.1186/s13011-018-0168-z)
Supplement: Supplementary file 2 — Checklist for Reporting Results of Internet E-Surveys (CHERRIES). (DOCX 23 kb) [file 13011_2018_168_MOESM2_ESM.docx]

| Checklist for Reporting Results of Internet E-Surveys (CHERRIES) | | |
| --- | --- | --- |
| Design | | |
|  | Survey design | Target population: Physicians in the US in full-time employment. Sample frame: Members of the general US population in full-time employment who are registered on Amazon’s Mechanical Turk (MTurk).  Survey items were based on the 571 descriptions of possible indicators of physician impairment or reasons to refer physicians to state physician health programs (PHPs) that are provided on the websites of 23 PHPs. See manuscript text for additional details. |
| Institutional review board (IRB) approval and informed consent process | | |
|  | IRB approval | The study was reviewed by the IRB of Vassar College, which determined it was exempt from full review. At the time the study was designed, the first author had no affiliations with any institution with an IRB, but had recently conducted another MTurk survey with protocol review by Vassar. |
|  | Informed consent | On MTurk, workers were told that the survey would take no more than 5 minutes to complete. (See Advertisement.) They were told, “We are conducting a survey about experiences and behaviors at work for an academic journal article. Your response will remain absolutely anonymous.” Before starting the survey, they were provided with an email to contact the first author, though the email did not reveal the identity of the authors.  MTurk provides all workers with detailed information regarding anonymity. Signing an informed consent agreement is required before MTurk workers can complete tasks. We did not attempt to document further their informed consent. |
|  | Data protection | Respondents were anonymized as soon as they agreed to participate and given a link to complete the survey on a separate website, psychsurveys.org. We used the “Complete Anonymity” setting on psychsurveys.org to ensure that no information regarding respondents’ IP addresses, or any other potentially identifiable information would be collected.  Through psychsurveys.org, we received information on respondents’ age, sex, race, and educational status. However, this information cannot be linked to any MTurk worker ID or IP address, and the data are reported only in aggregate. Participants’ responses and information are encrypted on MTurk by Secure Socket Layer (SSL) software. |
| Development and pre-testing | | |
|  | Development and testing | The survey instrument was novel and developed collaboratively by the authors. Two pilot versions of the survey were administered on June 2^nd^, 2017, and June 3^rd,^ 2017, with 8 and 10 participants respectively, to ensure technical functionality of the electronic questionnaire. These participants were excluded on MTurk from participating in the final survey. |
| Recruitment process and description of the sample having access to the questionnaire | | |
|  | Open survey versus closed survey | The survey was open to MTurk workers under the restriction that they resided in the US; had a task approval rating of at least 95%; had completed at least 1,000 prior tasks on MTurk; and were currently employed full-time, working at least 35 hours per week. |
|  | Contact mode | Only MTurk workers meeting these prescreening requirements could view and decide whether to participate in the study. MTurk workers are at least 18 years old. See above. |
|  | Advertising the survey | Our advertisement on MTurk read, “Title: Answer a survey about your experiences in work and other settings. Description: Tell us which descriptions apply to your experiences and behaviors at work and other settings.” The survey instructions appearing before workers accessed and started the survey read, “We are conducting a survey about experiences and behaviors at work for an academic journal article. Your response will remain absolutely anonymous.” Respondents were told that the survey would take no more than 5 minutes to complete; and that they would receive $0.30 electronically for their participation. |
| Survey administration | | |
|  | Web/e-mail | MTurk workers meeting prescreening requirements (see above) were provided a link to the survey, which was available on psychsurveys.org. |
|  | Context | On MTurk, workers browse tasks by title, keyword, reward, availability, and so on, to complete those that interest them. Previous research demonstrates that MTurk workers are internally motivated, and that raising compensation rates higher than this amount provided here is unlikely to increase survey response rates. |
|  | Mandatory/voluntary | Voluntary survey |
|  | Incentives | All participants completing the survey were paid $0.30 electronically. |
|  | Time/date | June 5^th^, 2017 to June 16^th^, 2017 |
|  | Randomization of items or questionnaires | Participants were assigned randomly to either the narrowly worded or broadly worded version of the survey. The 26 questions (either the narrowly worded versions or the broadly worded versions) on Page 2 appeared in the same order for all participants. |
|  | Adaptive questioning | Not used. |
|  | Number of items | 25 for both versions. (See Table 1.) |
|  | Number of screens (pages) | Page 1 (Demographic questions.)  Page 2 (25 questions, either narrowly worded, or broadly worded, depending on the survey version. [See Table 1.] An attention check question [either “Have you been born, and can you read this?” or “Have you ever been born, and can you read this?”] appeared as the twelfth question in order for each version of the survey, making 26 questions in total.)  Page 3 (Providing a unique code to enter on MTurk to receive compensation.) |
|  | Completeness check | All items for all participants were checked for completeness. |
|  | Review step | All 25 questions were administered on one page. Once the survey was completed, respondents could not access the survey again. |
| Response rates | | |
|  | Unique site visitor | 259 unique MTurk workers responded to the advertisement on MTurk by clicking the link that directed them to the first page of the survey (which collected demographic information) on psychsurveys.org |
|  | View rate (Ratio of unique survey visitors/unique site visitors) | N/A; It was not possible to view the site without at least viewing the first page of the survey (which collected demographic information) |
|  | Participation rate (Ratio of unique visitors who agreed to participate/unique first survey page visitors) | N/A; All visitors were provided information believed sufficient to make an informed consent; documentation of informed consent was not attempted |
|  | Completion rate (Ratios of users who finished the survey/users who agreed to participate) | 199/259 |
| Preventing multiple entries from the same individual | | |
|  | Cookies used | None. To identify participants, a unique log in code was provided. Once participants completed their surveys, they could not access their surveys again. |
|  | IP check | Not done, as we had no access to participants’ IPs for confidentiality reasons. We could not possibly connect their login codes, IDs, or any personally identifying information to IP addresses. |
|  | Log file analysis | Not done. |
|  | Registration | The survey was open. See above. |
| Analysis | | |
|  | Handling of incomplete questionnaires | Only surveys with all items completed were included in the results, as summarized in Table 3. Surveys with all items competed, but with all “No” answers on Page 2, including the attention check question (“Have you [ever] been born, and can you read this?”) were also not included in the results. |
|  | Questionnaires submitted with an atypical timestamp | None. |
|  | Statistical correction | None. |
|  | | |
